# Studying the Interfacial Properties of Carbon/Glass Hybrid Composites via the Nanoindentation Method

**DOI:** 10.3390/polym14142897

**Published:** 2022-07-16

**Authors:** Xin Jiang, Mingze Gao, Jing Zhu, Hongwei Ji, Fengchao Lang

**Affiliations:** 1 College of Energy and Power Engineering, Inner Mongolia University of Technology, Hohhot 010051, China; jiangxin@imut.edu.cn (X.J.); 664136612@imut.edu.cn (M.G.); 2 School of Science, Inner Mongolia University of Technology, Hohhot 010051, China; zhujing0451@126.com (J.Z.); 331181521@imut.edu.cn (H.J.); 3 The Department of 41, Dynamic Machinery Institute of Inner Mongolia, Hohhot 010010, China

**Keywords:** mechanical properties of the interface, carbon/glass hybrid, nanoindentation, fiber-to-fiber interaction

## Abstract

The mechanical properties of hybrid composite interfaces are critical in determining the overall properties of composite materials. To investigate the mechanical performance of hybrid composite interfaces, an accurate and efficient method must be developed. In this work, nanoindentation is used in this work to investigate the mechanical performance of the carbon/glass interface and the influence of the distance between carbon and the glass fibers on the modulus of the thermoset matrix. The results show that the interface sizes around the carbon and glass fibers are around 1.5 and 2.0 μm, respectively. The modulus around the carbon fibers is 5–11 GPa without the fiber effect, while that around the glass fibers is 4–10 GPa. The modulus of the matrix is not affected by the two types of fibers when the distance between them is greater than 4.5 μm.

## 1. Introduction

Hybrid composites are created by combining two or more types of fibers in a single matrix [1]. Hybrid composites exhibit better mechanical balance than non-hybrid composites. The aim of combining two different fibers into a composite is to retain the benefits of both fibers while overcoming some of their shortcomings. Replacing some of the central carbon fibers with cheaper glass fibers can significantly reduce the production cost of the material, while its flexural properties remain almost unaffected. The elongation of carbon fibers is low, while that of glass fibers is high. These fibers can be mixed and matched in many ways.

The initial objective of research on hybrid composites was to reduce material costs by substituting carbon fibers with less expensive fibers and raise the failure strain of hybrid composites. Extensive studies have been undertaken on the mechanical characteristics of hybrid composites, including their tensile properties [2,3,4,5,6,7,8], flexural properties [9,10,11], impact properties [12,13,14], and fatigue resistance capabilities [15,16], and it has been already established which configurations result in superior mechanical properties. The tensile modulus of hybrid composites has been shown to follow the linear rule of mixtures [2,3,4]. Values that deviate from this model can usually be attributed to the fiber volume fraction or the fiber orientation [3]. Intralayer unidirectional carbon/carbon fiber hybrids have a higher tensile modulus than their interlayer counterparts [4]. The difference is attributed to crimp, fiber misorientation, or measurement inaccuracies of the fiber volume fraction [5]. According to many researchers, the tensile strength of the hybrid effect is based on the bilinear rule of mixtures [6,7,8]. This viewpoint is based on a displacement-controlled test, which assumes an iso-strain for both high- and low-elongation fibers. Zhang et al. [4] discovered that improving fiber dispersion increases the ultimate tensile strength of glass/flax composites by 15%. 

The flexural performance of thermoset hybrid composites depends on the layup since longitudinal stress is zero in the neutral plane but increases linearly away from it. By changing the ply angles or the material type of each ply, additional opportunities can arise to improve the mechanical performance of hybrid composites. Dong et al. [9] demonstrated that carbon/glass fiber intralayer hybrids have flexural strengths that are 40% and 9% higher than those of full carbon and glass reinforcement composites, respectively. Carbon fibers increase the flexural strength when they are added to the tensile side of glass fiber reinforcement composites. The failure mechanism of glass fibers in carbon/glass hybrid composites under bending loads is primarily tension, while carbon fibers are destroyed by compression [10]. Several studies have shown that the flexural properties are lower than expected due to poor adhesion and interface quality [11,12,13]. 

The impact resistance of hybrid composites has been extensively studied since toughening is one of the most important phenomena linked with fiber hybridization and toughness is directly related to impact resistance. In a test with asymmetric layers of carbon/glass fibers, placing the carbon layers on the impacted side was found to increase the penetration resistance [14,15,16]. By adding the fibers with the highest energy absorption potential on the outside of the hybrid composite, it is possible to obtain hybrid composites that absorb more energy [12]. Hybrid composites are also expected to have a longer fatigue life and a lower fatigue life scatter compared with non-hybrid composites [17]. The fatigue lifetime of hybrid composites can be increased with respect to that of the high strain fibers reinforced composites due to the fact that the presence of high strain fibers delays the cracks propagations from the low strain fibers, reducing the likelihood of further high strain fiber failure and increasing the fatigue lifetime of the hybrid composites [18]. 

Previous studies have mainly focused on the mechanical properties of hybrid composites, but the influence of the interface on their properties has not been considered. The interface is a critical factor in determining the mechanical performance of composite materials [19,20,21]. The mechanical characteristics of the interface have a strong influence on the stress distribution, transfer, and microscopic mechanical properties of fiber-reinforced composite materials, consequently affecting their macro-mechanical performance. In addition, the interface also affects the internal damage, fracture accumulation, and fracture propagation in composite materials under stress, which determines durability of the composite [22,23]. By optimizing the interface quality of composite materials, both the fibers and the matrix may display superior mechanical properties, permitting the composite material to attain its maximum possible performance [24,25,26,27,28]. The distribution of the two fiber types plays a significant role in the production of hybrid composites. To obtain the optimal interface quality, the two fiber types must be adequately distributed. In this work, nanoindentation was used to explore the mechanical characteristics of the interface between carbon and glass fiber in hybrid composites. Nanoindentation has become a widely used technique for directly measuring the elastic modulus and hardness of both the composite materials and their constituents. Depending on the relative sizes of the nanoindentation equipment and the characteristic microstructural dimensions of the constituent materials, the results of the nanoindentation test reveal the effective properties of either the composite or its constituents [29,30]. The modulus for different distances between the carbon and glass fibers was studied in detail. 

## 2. Experimental Procedures 

### 2.1. Specimen Preparation 

**Figure 1:**
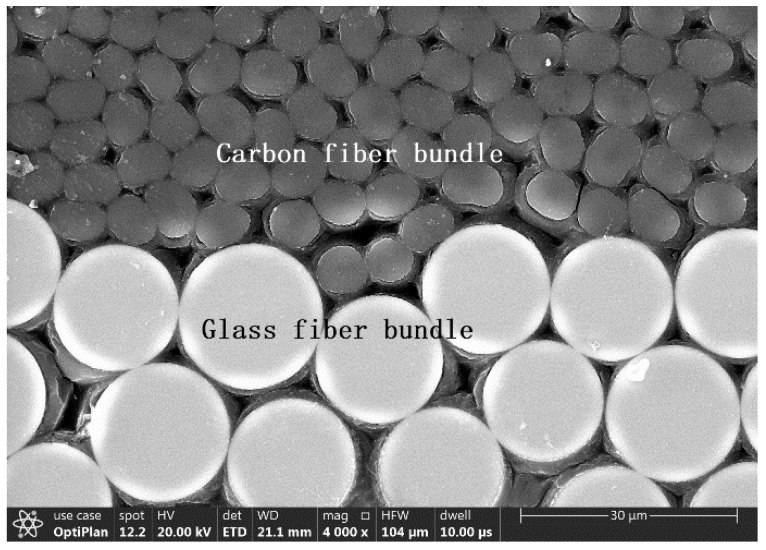
Image of the hybrid composite.

The carbon/glass fiber hybrid composite was fabricated using commercial carbon T-700-12K fibers and E-glass fibers (Guangwei composite materials Co., Ltd., Weihai, China). The diameters of the carbon and glass fibers were around 7 and 20 μm, respectively. The silane coupling agent was used to size the carbon and glass fibers to promote the fiber/matrix adhesion. The epoxy resin was NO.1-692-2A (Dongfeng chemical industry Co., Ltd., Guangzhou, China), and the hardener was NO.1-692-2B (BASF Co., Ltd., Guangzhou, China) they are mixed in a ratio of 10:3 by weight. The hybrid composite plate was made via a hand lay-up method at room temperature (23 °C) under a pressure of 0.6 MPa. The laminate was cured for 48 h under vacuum. The burn-off test revealed that the fiber volume fraction was around 65% A diamond saw was chosen to cut a sample with dimensions of 10×3×3 mm^3^ from the composite laminates perpendicular to the fiber direction. The cross-section of the sample was mechanically ground with 2000, 3000, and 5000 grit silicon carbide paper and then polished by SiO_2_ suspensions. The morphology of the sample was characterized via scanning electron microscopy (SEM, FEI, Apreo S LoVac, OR, USA), as shown in Figure 1.

### 2.2. Nanoindentation Measurements

The micromechanical properties of the hybrid composite were studied via nanoindentation. The nanoindentation method is commonly employed to analyze load–depth data by elastic contact theories. For shallow indents, meaningful modulus data have been achieved assuming linear elasticity during loading [31]. However, the most commonly adopted procedure relies on the analysis of the initial part of the unloading curve. The contact stiffness, *S* = d*P*/d*h*, is defined as the slope of the upper part of the unloading curve during the initial unloading stage. The relationship between the contact stiffness *S*, reduced modulus *E*_r_, and projected contact area *A* is as follows:

(1) $$E_{r}=\frac{\sqrt{\pi }}{2\beta }\frac{S}{\sqrt{A}}$$

For a Berkovich indenter, *β* = 1.034. *E*_r_ is defined as:

(2) $$\frac{1}{E_{r}}=\frac{1-\nu^{2}}{E}+\frac{1-\nu_{i}^{2}}{E_{i}}$$

where *E*_i_ = 1140 GPa and *ν*_i_ = 0.07 are the elastic modulus and Poisson’s ratio of the diamond indenter, respectively, and *E* and *ν* are the elastic modulus and Poisson’s ratio of the specimen, respectively. 

The hardness, *H*, is calculated according to the maximum load $P_{\max}$:

(3) $$H=\frac{P_{\max}}{A}$$

A Nano Indenter G200 (Agilent Technologies, Santa Clara, CA, USA) equipped with a Berkovich indenter was utilized to investigate the mechanical characteristics of the carbon and glass fibers, epoxy matrix, and fiber-matrix interface of the hybrid composite material. Displacement and load resolutions of 0.01 nm and 1 nN, respectively, were used in the nanoindentation test. The sample was indented at maximum displacements of 210 and 100 nm using the continuous stiffness measurement (CSM) method. A strain rate of 0.1 s^−1^ was applied. A small oscillating force of 2 nm in amplitude and 75 Hz in frequency was superimposed onto the loading cycle. The threshold for the thermal drift before the start of the experiments was set to 0.05 nm/s. 

## 3. Results and Discussion

### 3.1. Load vs Indentation Depth for the Different Phases 

To examine the mechanical characteristics of the carbon fibers, glass fibers, and resin matrix, indentation measurements with a maximum depth of 210 nm were performed in depth-control mode. An array of 20 × 20 nanoindentations was performed. Adjacent nanoindentations were separated by a distance of 2 µm to eliminate hardening effects and mutual influences.

**Figure 2:**
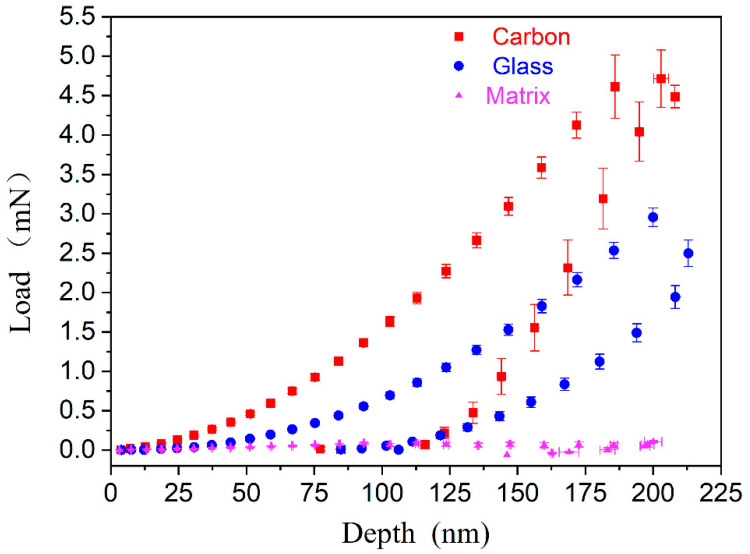
Load–depth curves for the three different phases.

Figure 2 depicts the load as a function of depth for each phase. Ten nanoindentation tests were conducted at each depth, which were then averaged to obtain final result. As shown in Figure 2, the maximum load on the carbon fiber (*F*_c_) is clearly larger than that on the glass fiber (*F*_g_) and matrix (*F_m_*): *F*_c_ ≈ 4.7 mN, *F*_g_ ≈ 3.0 mN, and *F_m_* ≈ 0.07 mN. In addition, the slope of the load–depth curves for the carbon fibers is higher than those obtained for the glass fibers and the matrix, which indicates that the carbon fibers are more resistant to deformation. The ratio of the unloading depth (*h_f_*) to the maximum depth (*h_max_*), *h_f_*/*h_max_*, was utilized to assess the extent of plastic deformation. The ratio was determined to be 0.38 for the carbon fibers, 0.53 for the glass fibers, and 0.73 for the matrix. This demonstrates that the value of *h_f_*/*h_max_* gradually increases from the carbon and glass fibers to the matrix, indicating that the residual indentation depth on the matrix is around two times that on the carbon fibers. No pop-in nor pile-up phenomena can be observed around the indentation, which may otherwise have influenced the results. Consequently, the measured indentation data should be correct and reliable.

### 3.2. Elastic Modulus and Hardness of the Different Phases

**Figure 3:**
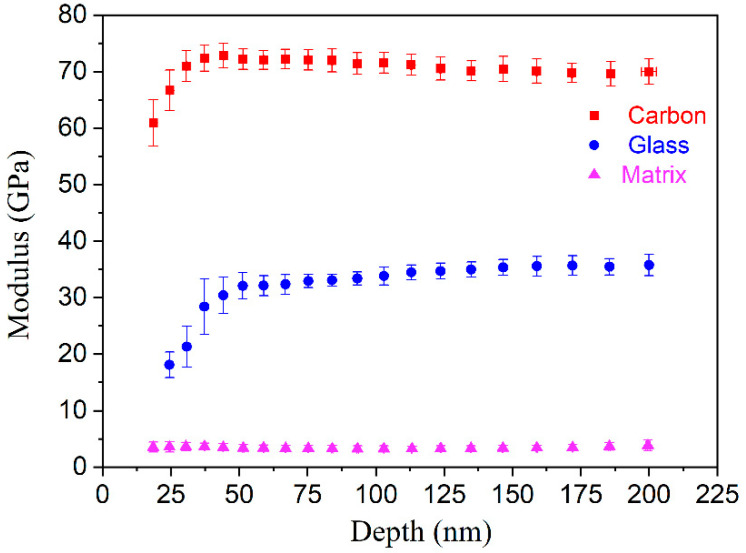
Modulus–depth curves for the three different phases.

Figure 3 shows the modulus as a function of the indentation depth for the different phases. The modulus values of the carbon fibers, glass fibers, and matrix are around 70, 35, and 3 GPa, respectively. Due to the limited resolution and the effect of the surface roughness, the indentation depths for the carbon and glass fibers are less than 50 nm; thus, the modulus data should be neglected. The modulus of the carbon fibers is around 1.6 times that of the glass fibers, but the residual depth of the glass fibers is approximately 1.4 times that of the carbon fibers. Carbon fibers have a higher stiffness than glass fibers, but their toughness is lower than that of glass fibers. Thus, by bringing these two different fiber types into a single composite, the advantages of both fibers can be retained, and several shortcomings can be overcome [32].

**Figure 4:**
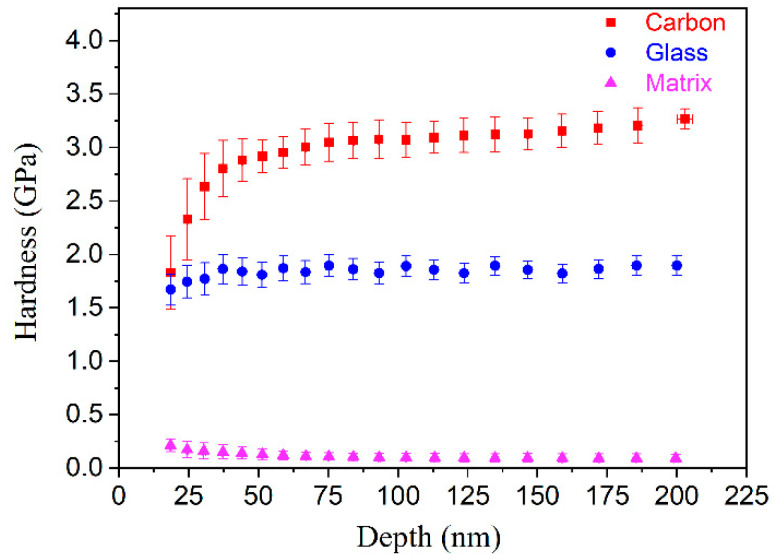
Hardness–depth curves for the three different phases.

The hardness of the three phases is shown in Figure 4. The hardness varies with depth in a manner similar to that of the modulus. As the indenter contacts the carbon fiber, the hardness steadily increases with increasing depth and eventually stabilizes at around 3.2 GPa. Throughout the indentation process, a constant hardness value of 1.7 GPa is maintained despite the hardness of the glass fiber. The matrix’s hardness is around 0.1 GPa. Carbon fiber is 1.9 times harder than glass fiber and 32 times harder than the matrix.

### 3.3. Interface around the Carbon and Glass Fibers

Due to the small size of the interface, it is easy for the indenter to contact the edge of the fibers during the indentation process, which has a direct effect on the measurement of the modulus of the interface [33]. Considering the triangular prism shape of the Berkovich indenter, the relationship between the indentation depth, *h*, and the distance, *d*, from the outermost edge to the center of the indentation can be expressed as:

(4) h=d/3.7

When the distance between the center of the indentation and the fiber edge is greater than 3.7 *h*, the indenter does not contact the fiber edge and the fiber reinforcing effect can thus be neglected. 

**Figure 5:**
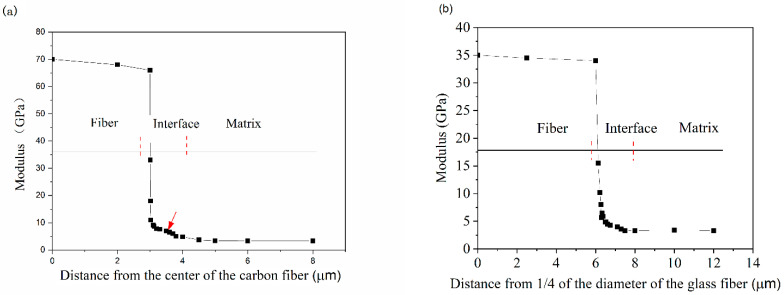
Interface around the carbon and glass fibers: (**a**) carbon fibers and (**b**) glass fibers.

Figure 5a depicts the variation in modulus at different positions around the carbon fiber. When the indentation test proceeds from the carbon fiber to the matrix across the interface, the modulus decreases dramatically owing to an increase in the volume deformation percentage of the matrix. A sudden drop in modulus occurs, suggesting that the indenter has come into contact with the fiber edge during the test. The modulus plateau, which is indicated by the red arrow in Figure 5a, is within 1.5 μm of the fiber edge, demonstrating that the modulus in this region is unaffected by the fiber and matrix effects. These intermediate modulus values indicate that a pure interface zone exists between the fibers and the matrix. Therefore, the size of the interface around the carbon fiber is around 1.5 μm, the modulus varies from 11 to 5 GPa, and its average value is 7 GPa. The tests were conducted at a distance greater than 1.5 μm from the fiber edge, and the corresponding modulus is 3.4 GPa, which indicates that the measurement data were collected from the matrix.

The modulus variation at different positions along the glass fiber is shown in Figure 5b. In the figure, the origin point corresponds to 1/4 of the fiber diameter (20 μm), whereas the length of the fiber edge is 5 μm. The changes in the modulus around the glass fiber as a function of distance are identical to those around the carbon fiber. The interface size of the glass fiber is slightly greater than that of the carbon fiber, being around 2 μm. In the range of distances between 0.025 and 2.0 μm from the fiber edge, the modulus decreases from 10 to 4 GPa and its average value is around 6 GPa. When the distance from the fiber edge exceeds 2 μm, the modulus reaches the value of the matrix. 

### 3.4. Influence of Different Fiber Spacings

**Figure 6:**
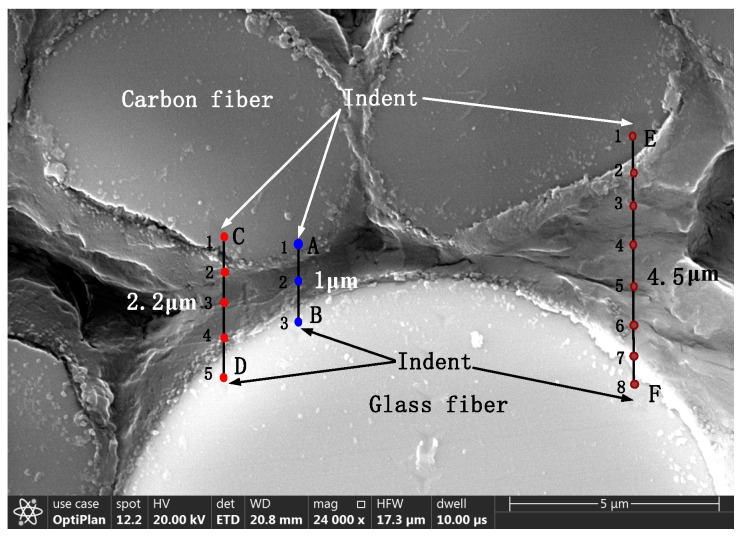
SEM image illustrating the positions of the different indentation measurements.

To study the interaction between carbon and glass fibers with different spacings, three different distances were selected, as shown in Figure 6. The distances between the AB, CD, and EF segments are 1, 2, and 4.5 µm, respectively. The influence of different spacings between the carbon and glass fibers was investigated at a maximum indentation depth of 100 nm. Different numbers of indentations were performed along the three segments, as shown in Figure 6. The indents on the surface of the glass fiber are indicated by the black arrows. The distance between two adjacent indentations is 1 μm to avoid any possible interaction.

Three points were tested along the AB segment: points 1 and 3 correspond to positions on the carbon and glass fibers, respectively, and their indentation values are 68 and 34 GPa, respectively; the modulus at point 2 in the middle of the AB segment is 13 GPa, which is 4.3 times that of the matrix (3 GPa). Therefore, when the distance between the two fibers is small, the modulus of the matrix between the two fibers increases due to the influence of the fibers. 

**Figure 7:**
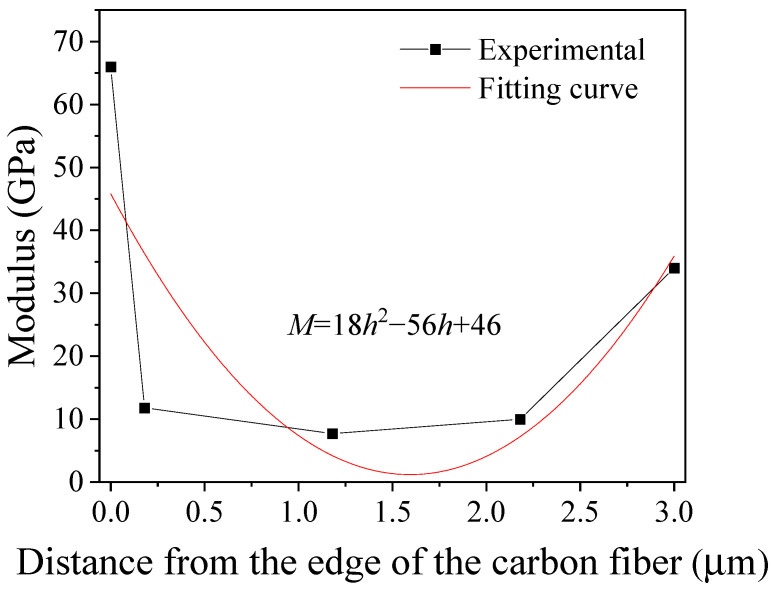
Experimental (symbols) and fitted (curve) modulus along the EF segment in Figure 6.

When the distance between the edge of the carbon fiber and the edge of the glass fiber is 2.2 µm, except for the two measurement points corresponding to the fibers themselves, there are three measurement points between the two fibers, as shown in Figure 7. The modulus along the CD segment is shown in Figure 7. The modulus decreases as the distance from the edge of the carbon fiber increases and remains almost constant in the region between the two fibers. The minimum modulus is 7.7 GPa, which is 2.6 times that of the matrix (3 GPa). By fitting these experimental data, the relationship between the modulus and the distance from the edge of the carbon fiber can be obtained. This relationship is $M=18h^{2}-56h+46$, where *M* is the modulus, and *h* is the distance from the edge of the carbon fiber. There is a large deviation between t the experimental data and the fitting curve in the region close to the carbon fiber, which is mainly caused by the sharp decrease in the experimentally measured modulus.

**Figure 8:**
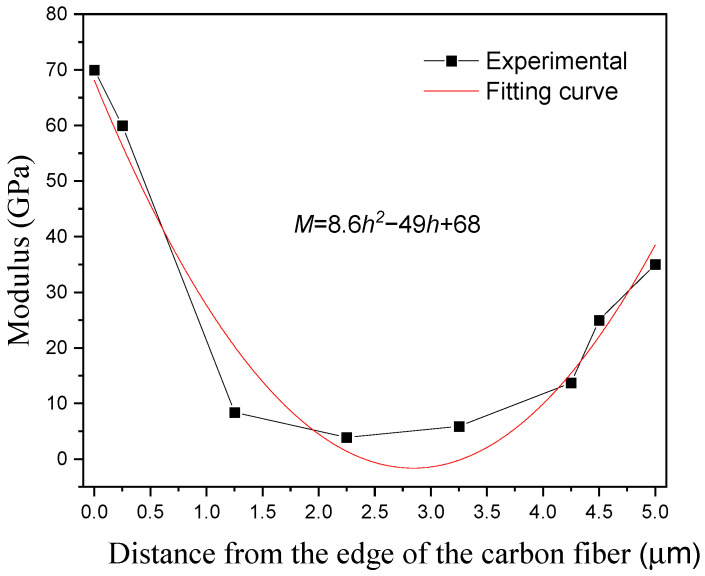
Experimental (symbols) and fitted (curve) modulus along the CD segment in Figure 6.

The modulus along the CD segment is shown in Figure 8. It can be seen from the figure that as the distance between the two fibers increases, the modulus gradually decreases, and when the distance between the two fibers is greater than 4.5 µm, their mutual influence can be ignored. The widths of the interface regions around the carbon and glass fibers are 1.5 and 2 µm, and the distance over which there exists a mutual influence is slightly larger than the sum of their respective interface widths. The modulus in the region between the carbon and glass fibers satisfies the following parabolic trend: $M=8.6h^{2}-49h+68$. The modulus at the middle of this distance is the same as that of the matrix, indicating that when the distance is greater than 4.5 μm, the mechanical properties of the matrix are not affected by the fibers on either sides.

## 4. Conclusions

The mechanical characteristics of carbon/glass hybrid composites were studied using the nanoindentation method. The indentation results for the carbon fibers, glass fibers, and interface as well as those at different distances between the carbon and glass fibers were analyzed to understand how the modulus changes. The conclusions we reached are as follows:

The modulus values of the carbon fibers, glass fibers, and epoxy matrix were determined to be 70, 35, and 3 GPa, respectively, whereas the corresponding hardness values were 3.2, 1.7, and 0.1 GPa, respectively. The modulus of the interface between the carbon fibers and the matrix could be evaluated after taking into account the fiber-bias effect, and the value was estimated to be 5–11 GPa. The interface size was found to be around 1.5 μm. The size of the interface between the glass fibers and the matrix was around 2.0 μm, and the corresponding modulus was 4–10 GPa.

When the distance between the carbon and glass fibers was greater than 4.5 μm, the modulus of the matrix was not affected by the two fiber regions. The modulus in the region between the fibers was found to satisfy a parabolic trend. When the distance from the edge of the carbon fibers was around 1 μm, the matrix modulus was greatly affected by the fibers, and the modulus of the matrix was found to be approximately 4.3 times that of the matrix without the effect of the fibers.

## Data Availability

There are no linked research datasets for this submission.
